# Continuous adductor canal block provides better performance after total knee arthroplasty compared with the single-shot adductor canal block? An updated meta-analysis of randomized controlled trials

**DOI:** 10.1097/MD.0000000000022762

**Published:** 2020-10-23

**Authors:** Rongguo Yu, Haiyang Wang, Youguang Zhuo, Dongxin Liu, Chunling Wu, Yiyuan Zhang

**Affiliations:** a Department of Orthopedics, Fuzhou second Hospital Affiliated to Xiamen University, Fujian; b Hebei North University, Handan Central Hospital Affiliated to Hebei North University, China.

**Keywords:** adductor canal, analgesia, CACB, meta-analysis, nerve block, SACB, total knee arthroplasty

## Abstract

**Background:**

Adductor canal block (ACB) has emerged as an attractive alternative for femoral nerve blocks (FNB) as the peripheral nerve block of choice for total knee arthroplasty (TKA), preserving quadriceps motor function while providing analgesia comparable to FNB. However, its optimal application for TKA remains controversial. The objective of this meta-analysis was to compare continuous-injection ACB (CACB) vs single-injection ACB (SACB) for postoperative pain control in patients undergoing TKA.

**Methods:**

This study attempts to identify the available and relevant randomized controlled trials (RCTs) regarding the analgesic effects of CACB compared to SACB in patients undergoing TKA according to electronic databases, including PubMed, Medline, Web of Science, EMbase, and the Cochrane Library, up to September 2019. Primary outcomes in this regard included the use of a visual analogue scale (VAS) pain score with rest or activity, while secondary outcomes were cumulative opioid consumption, length of hospital stay (LOS), complications of vomiting and nausea, and rescue analgesia. The corresponding data were analyzed using RevMan v5.3.

**Ethical review:**

Because all of the data used in this systematic review and meta-analysis has been published, the ethical approval was not necessary

**Results:**

This research included 9 studies comprised of 739 patients. The analyzed outcomes demonstrated that patients who received CACB had a better at rest-VAS scores at 4 hours (*P* = .007), 8 hors (*P* < .0001), 12 hours (*P* < .0001), 24 hours (*P* = .02), mobilization-VAS score at 48 hours (*P* < .0001), and rescue analgesia (*P* = .03) than those who underwent SACB. Nevertheless, no significant differences were present between the 2 strategies in terms of pain VAS scores 48 hours at rest (*P* = .23) and 24 hours at mobilization (*P* = .10), complications of vomiting and nausea (*P* = .42), and length of hospital stay (*P* = .09).

**Conclusion:**

This meta-analysis indicated that CACB is superior to SACB in regard to analgesic effect following TKA. However, due to the variation of the included studies, no firm conclusions can be drawn. Further investigations into RCT are required for verification.

## 1 Introduction

TKA is regarded as an effective treatment in the treatment of end-stage knee osteoarthritis.^[1,2]^ Reports analyzed from different counties assert that, even with conservative estimates, the increased use of knee replacement will continue,^[3,4]^ which is estimated to grow 12% by 2025.^[5]^ Postoperative pain after knee surgery is increasingly more common in these patients.^[6]^ Studies suggest that over 60% of patients^[7,8]^ experience moderate to severe postoperative pain,^[9,10]^ with many associated adverse effects.^[8]^ Poor pain control has resulted in prolonged hospital stay, reduced patient satisfaction, delayed convalescence, and ambulation.^[11–14]^

It is vital that patients with post-TKA receive effective postoperative analgesia, which improves their levels of satisfaction. To relieve pain and increase improve clinical outcomes of TKA, patient controlled intravenous analgesia (PCIA), FNB, intravenous analgesia, and epidural analgesia are the most commonly employed analgesic modalities.^[15,16]^ PCIA requires a high dose of opioids, which may lead to additional adverse events. Moreover, those who receive epidural analgesia may have a higher rate of urinary retention and hypotension.^[17]^ Furthermore, FNB may possess better pain-relieving functions compared to patient-controlled analgesia (opioids).^[18,19]^ As a peripheral nerve block, FNB is a well-established analgesia strategy and is considered to be the standard in postoperative TKA pain management.^[15,20,21]^ However, patients that receive FNB usually suffer from a marked reduction in quadriceps muscle strength,^[22,23]^ increasing their risk for postoperative fall.^[24]^

ACB serves as another analgesic technique, which has been rapidly developed in recent years due to its lower incidence of complications and higher success in pain control post-TKA, according to the latest studies.^[25–27]^ ACB has emerged as an alternative to FNB after TKA.^[27–29]^ ACB lessens the amount of analgesia around most of the quadriceps muscle, accelerating physiotherapy^[30]^ and reducing falls after TKA.^[31]^ ACB is increasingly being considered over other analgesic options for use in patients receiving TKA.^[29,32]^

However, its optimal strategy of use remains unknown. Many clinicians perform a single shot ACB, however, others have reported success using a continuous injection for over 24 hours or 48 hours following surgery. Currently, the disadvantages of a single infusion over a continuous infusion are debatable. Critics have debated that similar analgesic properties exist between the 2 due to the length of analgesia being over 12 hours. Simultaneously, the maintenance and insertion of continuous catheters are associated with the excessive consumption of human resources. Additionally, a controversy was reported in that patient rehabilitation and physiotherapy may be adversely affected by continuous postoperative infusion.^[28]^

Choosing a safe and effective analgesic strategy is necessary for the acceleration of patient recovery in surgery. Consequently, this study aims to determine whether CACB is a better strategy for postoperative pain control and rehabilitation for limb function compared to SACB. Furthermore, the optimal application of ACB following TKA is also discussed.

## 2 Materials and methods

### 2.1 Search strategy

This study was performed by adhering to the 2009 PRISMA (Preferred Reporting Items for Systematic Reviews and Meta-Analysis) guidelines.^[33]^ We identified randomized controlled trials up to September 2019 by searching databases including PubMed, Medline, Web of Science, EMbase, and the Cochrane Library using the following terms: (total knee replacement or total knee arthroplasty) and (adductor canal block or saphenous nerve block). Additionally, the reference lists of review articles, additional trials, and other reports were also included by manual search.

### 2.2 Inclusion and exclusion criteria

RCTs were included in our meta-analysis if they met the following PICOS (patients, intervention, comparator, outcome, study design) criteria:

1. Patients: some had received TKA for the first time.
2. Intervention: patients received SACB analgesia after TKA.
3. Comparator: patients received CACB analgesia after TKA.
4. Outcomes: cumulative morphine consumption, complications of vomiting and nausea, VAS score at rest and movement, rescue analgesia, and LOS.
5. Study design: RCTs.

Exclusion criteria included non-randomized trials, review articles, quasi-randomized trials, cadaver studies, comments, protocols, letters, editorials, guidelines, surgical registries and review papers, reports involving bilateral TKA, revision knee arthroplasty and articles containing insufficient outcome data. Discrepancies were reconciled through discussions.

### 2.3 Study selection

The identification of studies followed the predefined eligibility criteria. After discarding any duplicates, 2 researchers independently screened the abstracts, and the titles and abstracts of all studies ascertained using the employed the search strategy were collected, abandoning those that were ineligible. The full article was assessed if its eligibility could not be determined. Any disagreements were resolved through discussion among researchers.

### 2.4 Data extraction

Two authors retrieved the relevant information independently from the articles using a standard data extraction form. The collected data included population, age, author, study design, sample size, publishing date, gender, dosages, and type of analgesia, and type of interventions. Primary outcomes included the visual analogue scale (VAS) pain score at rest and mobilization (determined via patient interviews at 4, 8, 12, 24, and 48 hours post-TKA; 0 = no pain and 10 = worst imaginable pain). Additionally, secondary outcomes included complications of vomiting and nausea (If the study reported the frequency of vomiting and nausea events or requiring additional other treatments by doctor relevant this aspect, the data was extracted in our research.), cumulative total morphine consumption [all opioids given were converted to morphine equivalents (Meq) at 48 hours], rescue analgesia and the length of stay (LOS) in hospital (days). If necessary, the corresponding authors of the included articles were contacted to confirm that the information aligned with our criteria. Disagreements were reconciled through discussion.

### 2.5 Quality assessment and risk of bias

Six respects were taken into account to evaluate the risk of bias through random sequence generation, allocation concealment, blinding of assessors, incomplete data, blinding of participants and personnel, selective reporting and other biases.^[34]^ Two reviewers independently assessed the quality of the included studies with the use of the Cochrane Collaboration tool (domain-based risk-of-bias tables).^[35]^ Each item was required to be measured as “Unclear” (unclear risk of bias), “Yes” (low risk of bias), or “No” (high risk of bias). The risk of bias summary (Fig. 2) and the risk of bias graph (Fig. 3) were obtained using Review Manager (RevMan) version 5.3 (The Nordic Cochrane Center, The Cochrane Collaboration, 2009, Copenhagen, Denmark). In case of divergence, a consensus was reached via discussion between more than 2 authors.

### 2.6 Statistical analysis

Review Manager for Windows (Version 5.3) was used to finish the meta-analyses. For dichotomous outcomes, the results were presented as relative risks (RR) with a 95% CI. Continuous variable outcomes were assessed using the standard mean difference (SMD) or mean difference (MD) with a 95% confidence interval (CI). The Chi-Squared test was performed to evaluate the heterogeneity of studies according to the values of *P* and *I*^2^. When *I*^2^ < 50% and *P* > .1, the fixed-effects model was used. Otherwise, the random-effects model utilized for the meta-analysis, which was performed to investigate the source of heterogeneity by the subgroup analysis.

### 2.7 Study selection and characteristics of the selected studies

**Figure 1:**
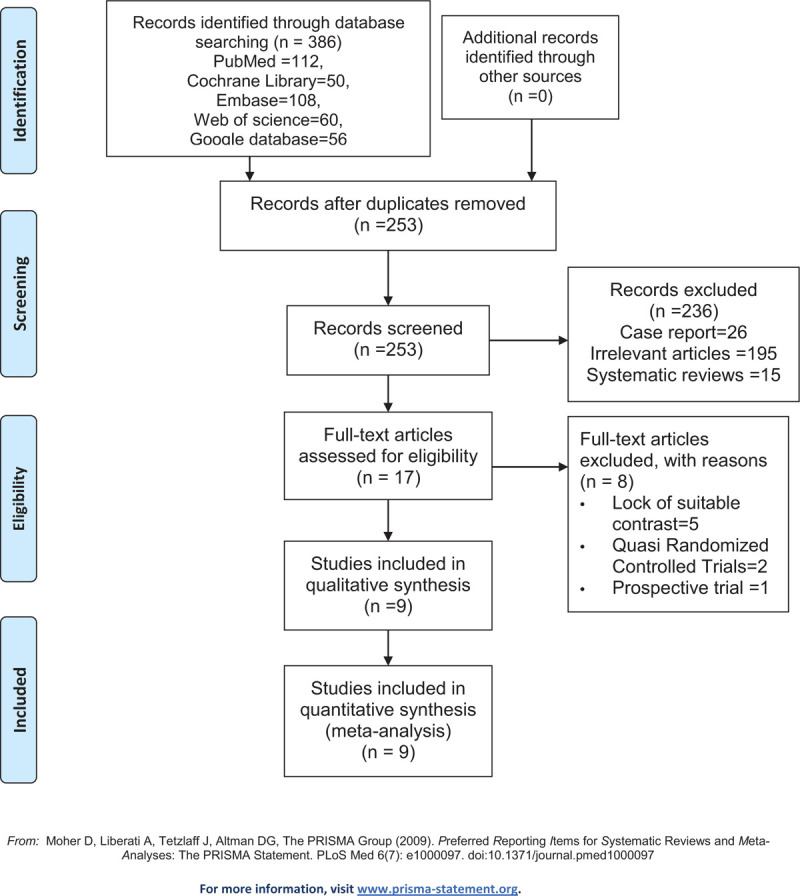
The PRISMA flow diagram detailing our literature search.

In the initial search, a total of 386 studies were identified from the electronic databases (PubMed = 112, Cochrane Library = 50, EMBASE = 108, Web of Science = 60, Google database = 56). All of the collected studies were then input into Endnote X7 (Thomson Reuters Corp., USA) software to exclude any duplicates. The 253 studies were reviewed, after which 133 papers were discarded according to the inclusion criteria at the title and abstract levels. Additionally, as 1 study was a duplicate, the most recently published paper was only considered. Two articles^[36,37]^ highly aligned with the requirements, however, only the abstracts were able to be collected, rather than the full texts. Ultimately, 9 clinical studies comprised of 739 patients (CACB group = 371, SACB group = 368) were included in the meta-analysis.^[38–46]^ The sample size of the included studies ranged from 22 to 63. The flow diagram pertaining to the included studies are in Figure 1, and the general characteristics of the included studies can be shown in Table 1. Additionally, the analgesia intervention protocol of the RCTs included in the meta-analysis is illustrated in Table 2.

**Table 1:**
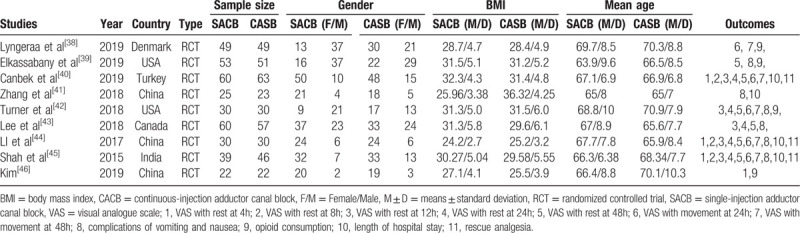
General characteristics of RCTs included in the meta-analysis.

**Table 2:**
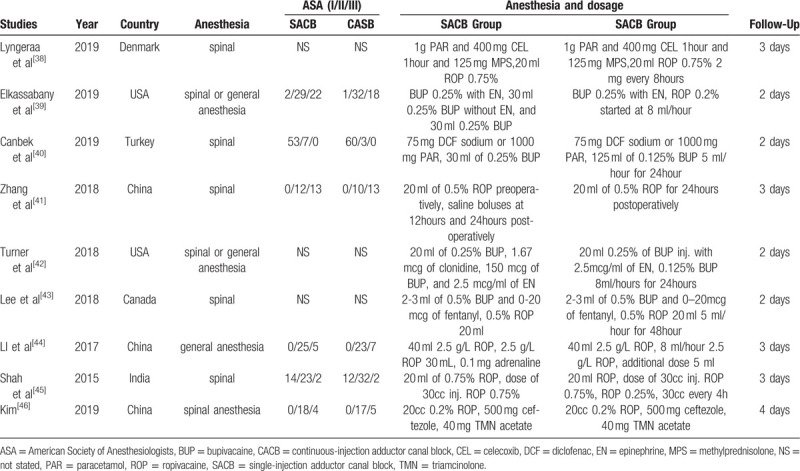
Study anesthesia intervention protocol of RCTs included in the meta-analysis.

### 2.8 Quality assessment and risk of bias

**Figure 2:**
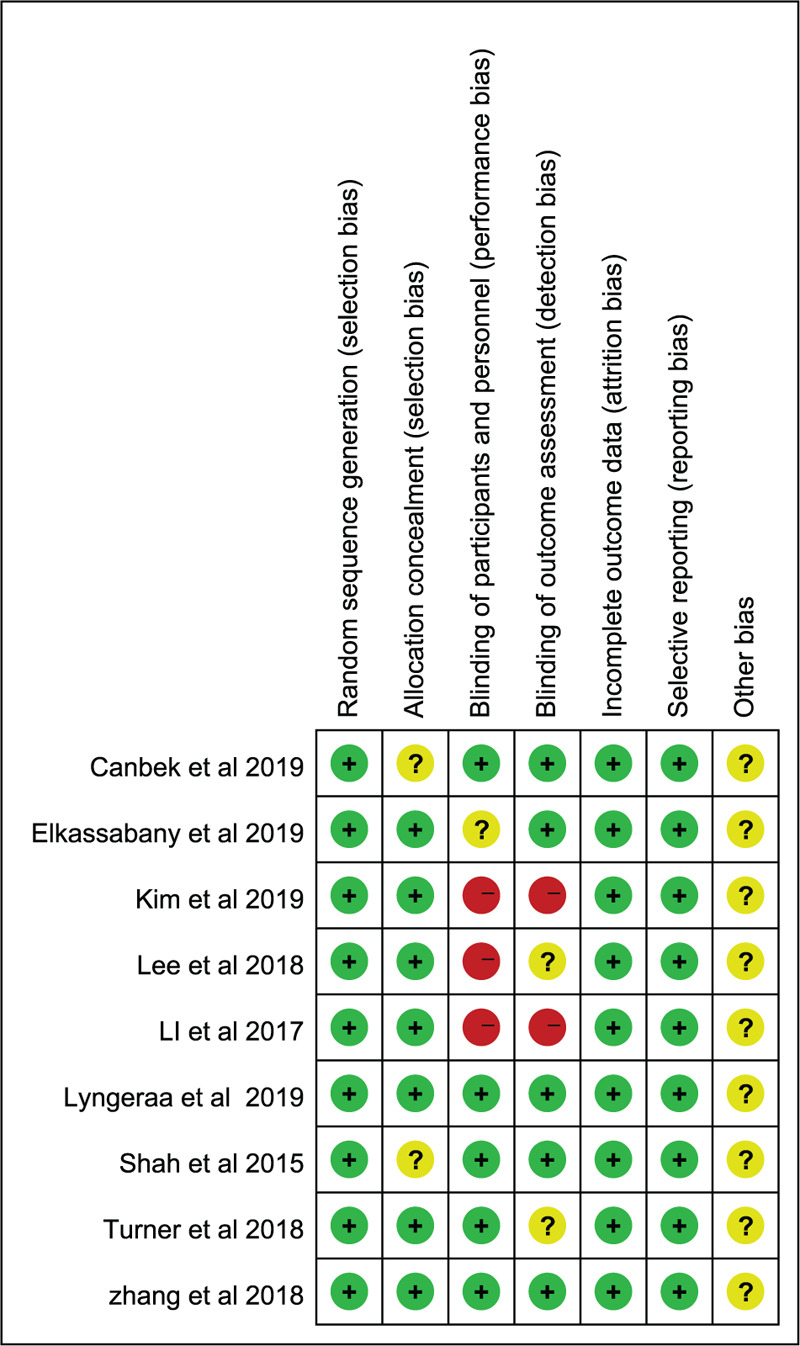
The risk of bias summary of the included studies. (+ represents yes; – represents no?; represents not clear).

**Figure 3:**
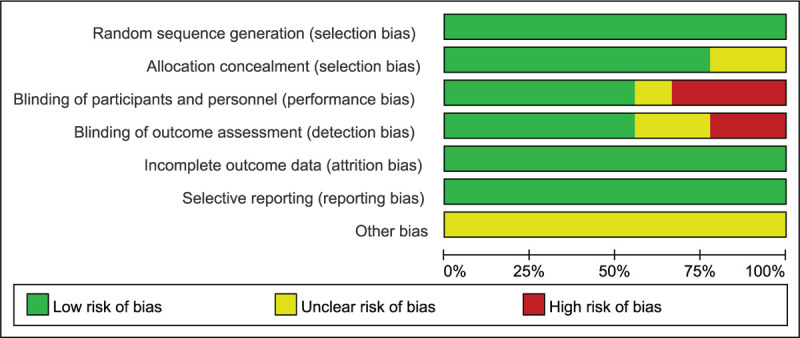
The risk of bias graph of the included studies.

The methodological quality of all included RCTs was evaluated according to the Cochrane Handbook for Systematic Reviews of Interventions. Correspondingly, 9 RCTs discussed adequate randomization techniques like random number lists,^[42]^ computer-generated block randomization,^[38–41,43–46]^ and sealed random number envelope.^[38,39,41–43,46]^ Allocation concealment was described in 2 trials^[38,42]^ but was unclear in 7 trials.^[39–41,43–46]^ The blinding of personnel and participants were mentioned in 3 trials^[38,42,45]^ but was unclear in 6 trials.^[39–41,43,44,46]^ Studies considered low risk for attrition bias with complete data were also included. Figures 2 and 3 summarized the specific risk of bias in methodological quality for the eligible RCTs. Publication bias was assessed by using a funnel plot diagram (Fig. 10 A-E).

## 3 Results

### 3.1 Primary outcomes

#### 3.1.1 VAS score at rest

**Figure 4:**
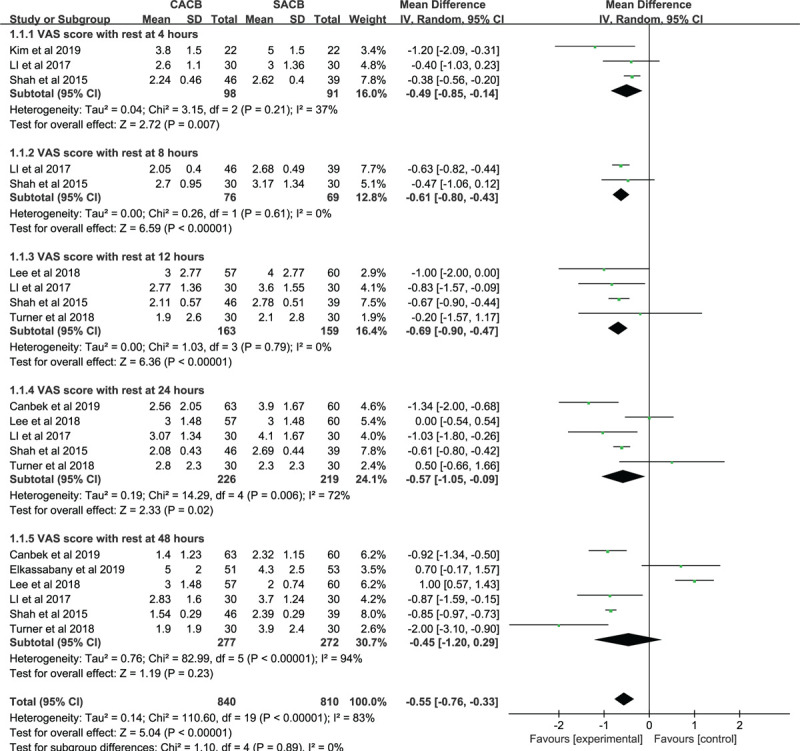
Forest plots of the pain VAS score with rest between CACB group and SACB group after TKA.

Only 3 studies (189 patients) reported the complications of VAS scores at rest within 4 hours after TKA. A significant difference was observed between the SACB and CACB groups (MD = −0.49; 95% CI: −0.85–−0.14; *P* = .007; Fig. 4). Two studies comprised of 145 patients reported VAS scores at rest at 8 hours postoperatively, and significant differences were exhibited between the2 groups (MD = −0.61; 95% CI: −0.80–−0.43; *P* < .0001; Fig. 4). Three hundred twenty two knees from 4 studies involved reported the VAS score at 12 hours at rest. This meta-analysis showed significant differences between the SACB and CACB groups (MD = −0.69; 95% CI: −0.90–−0.47; *P* < .0001; Fig. 4). Data from 5 studies on 445 patients were available to examine the pain score during rest on postoperative at 24 hours. There was a significant difference between the SACB and CACB groups (MD = −0.57, 95% CI: −1.05 –−0.09, *P* = .02; Fig. 4). The VAS score during rest was reported by 5 studies, including 549 patients at 48 hours. There was significant difference between the SACB and CACB groups (MD = −0.45; 95% CI: −1.20–0.29, *P* = .23; Fig. 4).

#### 3.1.2 VAS Score with movement

**Figure 5:**
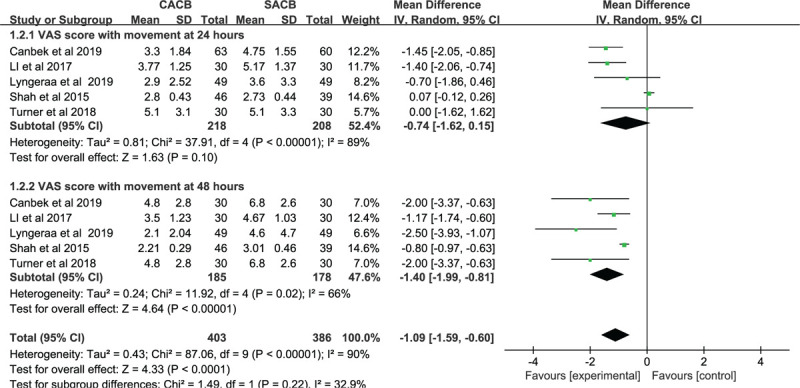
Forest plots of the pain VAS score with movement between the CACB group and SACB group after TKA.

Five studies with 426 patients reported the pain score during movement at 24 hours after postoperative. It showed no statistical significance between the 2 groups (MD = −0.74, 95% CI: −1.62 to 0.15, *P* = .10; Fig. 5). Five studies involving 363 patients showed the VAS scores during movement at 48 hours, and the important statistical difference was shown between the 2 groups (MD = −1.40, 95% CI: −1.99–−0.81, *P* < .00001; Fig. 5).

### 3.2 Secondary outcomes

#### 3.2.1 Complications of vomiting and nausea

**Figure 6:**
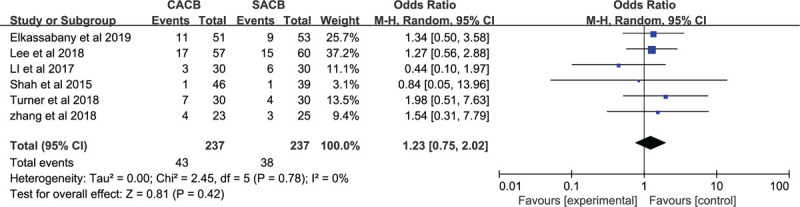
Forest plots of the complications of vomiting and nausea between CACB group and SACB group after TKA.

Only 6 studies reported the complications of vomiting and nausea. No significant difference in nausea or vomiting was found between the 2 groups (odds ratio = 1.54; 95% CI: 0.31–7.79; *P* = .42, Fig. 6).

#### 3.2.2 Cumulative opioid consumption

**Figure 7:**
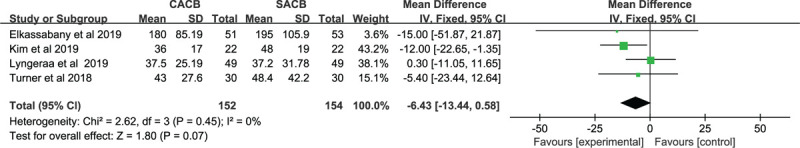
Forest plots of the cumulative opioid consumption within 48 hours between CACB group and SACB group after TKA.

The number of 4 studies (306 patients) provided related data on cumulative opioid consumption. The pooled data showed no significant difference between the CACB and SACB groups at 48 hours (MD = −6.43; 95% CI: −13.44–0.58; *P* = .07; Fig. 7).

#### 3.2.3 LOS

**Figure 8:**
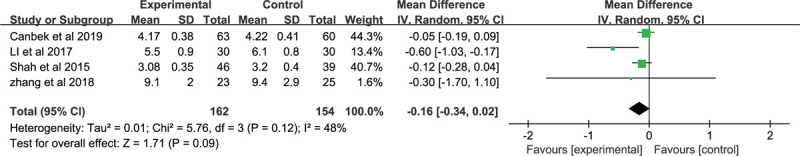
Forest plots of the length of hospital stay between the CACB group and SACB group after TKA.

LOS was reported in 4 studies, and a total of 316 patients were involved in the meta-analysis. The data showed no significant difference between the CACB and SACB groups (MD = −0.16; 95% CI: −0.34–0.02; *P* = .09, Fig. 8).

#### 3.2.4 Rescue analgesia

**Figure 9:**
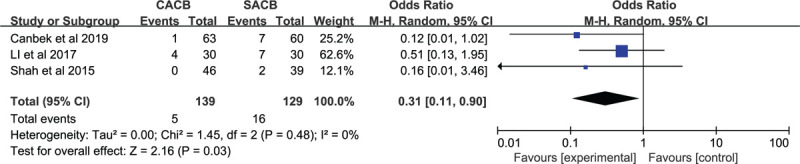
Forest plots of the rescue analgesia between the CACB group and SACB group after TKA.

Only 3 studies (268 patients) reported about the rescue analgesia. It found significant statistical significance in rescue analgesia between the 2 groups (MD = 0.31; 95% CI: 0.11–0.90; *P* = .03, Fig. 9).

#### 3.2.5 Publication bias

**Figure 10:**
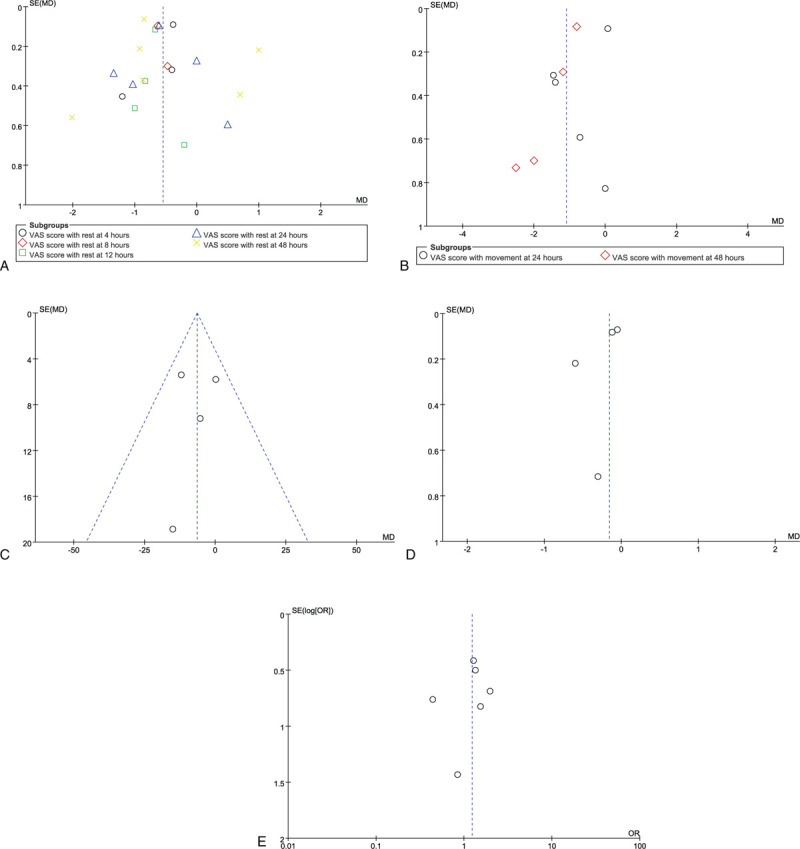
A. Funnel plot of publication bias for the pain score with rest between CACB group and SACB group after TKA. There was symmetry, suggesting that there was not a significant publication bias. B. Funnel plot of publication bias for the pain score with movement between the CACB group and SACB group after TKA. There was symmetry, suggesting that there was not a significant publication bias. C. Funnel plot of publication bias for the cumulative opioid consumption within 48 hours between CACB group and SACB group after TKA. There was symmetry, suggesting that there was not a significant publication bias. D. Funnel plot of publication bias for the length of hospital stay between CACB group and SACB group after TKA. There was symmetry, suggesting that there was not a significant publication bias. E. Funnel plot of publication bias for nausea or vomiting between the CACB group and the SACB group after TKA. There was symmetry, suggesting that there was not a significant publication bias.

Five funnel plots based on the VAS score at rest, VAS score with movement, complications of vomiting and nausea, cumulative opioid consumption, length of hospital stay and rescue analgesia were used to assess publication bias, which demonstrated minimal asymmetry with few outliers, indicating minimal evidence of publication bias (Fig. 10 A–E.)

## 4 Discussion

In recently published extensive comments, ACB was found to protect quadriceps strength to facilitate early mobilization and confer a degree of analgesic effect similar to that of FNB.^[47]^ Achieving a balance between muscle strength and analgesia was one of the purposes of pain management post-TKA. As ACB is a purely sensory block, the motor function of only the medial rectus is affected.^[32,48]^ Recent studies have shown that FNB initially reduced quadriceps strength by 49%, however, quadriceps strength was decreased by 8% by ACB.^[49]^ The study reported that ACB could be treated as a continuous infusion or single-shot injection.^[50]^ But there are no definite conclusions that may be made regarding which is better.

To our knowledge, this is not the first meta-analysis of RCTs comparing the efficacy and safety of SACB with CACB to get command of pain after TKA. However, the authors believe that this meta-analysis is more complete than the previous meta-analysis published by Zhang et al.^[51]^ Our study included 5 recent high-quality RCTs,^[38–41,46]^ thus decreasing publication bias and statistical bias. We extract data more objectively, bringing about more precise conclusions. Consequently, these factors strengthen the quality of this studys findings. Outcomes showed that patients who received CACB had a better VAS score with rest at 4 hours (*P* = .007), 8 hours (*P* < .0001), 12 hours (*P* < .0001), 24 hours (*P* = .02), mobilization 48 hours (*P* < .0001), and rescue analgesia (*P* = .03) than those that underwent SACB. However, no significant differences between the 2 strategies in pain scores 48 hours at rest (*P* = .23) and 24 hours at mobilization (*P* = .0009), complications of vomiting and nausea (*P* = .42), and length of hospital stay (*P* = .09). Therefore, CACB may now serve as a better analgesia strategy after TKA.

### 4.1 VAS score

Several studies have reported the efficacy of ACB in postoperative analgesia following arthroscopic procedures or TKA.^[29,52–57]^ Severe pain following TKA, particularly during early physiotherapy and mobilization, may sustain through 48 hours after surgery.^[58,59]^ Interestingly, few articles reported that the duration of analgesia from SACB is typically 12 to 24 hours,^[60]^ but maybe as long as 48 hours,^[59]^ with varying efficacy. Pain intensity was evaluated as VAS scores at 481,224 and 48 hours after TKA. Furthermore, the comprehensive analysis showed that the CACB group and SACB groups demonstrate noticeable differences in VAS scores at static positions at 4 hours (*P* = .007), 8 hours (*P* < .0001), 12 hours (*P* < .0001), 24 hours (*P* = .02) or mobilization at 48 hours (*P* < .00001). Meanwhile, the present study shows that TKA patients who receive CACB can acquire similar VAS scores with rest at 48 hours (*P* = .23) or mobilization at 24 hours (*P* = .10) than those treated with SACB.

The VAS score is a subjective scale that is easily influenced by individual factors. We implemented a subgroup analysis according to the RCTs. Accordingly, the CACB groups may share associations with local analgesia in prolonging analgesic duration compared to SACB groups.^[61]^ Therefore, providing adequate analgesia for a longer period may be expected when using continuous infusion catheters.

VAS scores with mobilization at 48 hours demonstrates a certain advantage, which is consistent with that of the other 2 articles regarding the numeric rating scale (NRS) score. Although no other changes in strategies, Turners et al^[42]^ reported critical differences that appeared apparently at 42 hours in the NRS score when the CACB deviate to the SACB group. It was observed that the continuous-injection group keeps past the duration of the single-shot group after 36 hours. Kim et al^[46]^ reported that the NRS score of SACB may be better at 48 hours after surgery (*P* < .05) compared to CACB. However, researchers may disagree on a fixed parameter for dosage as well as the timing of ACB. According to Jain and Shah,^[45]^ it was filled into the adductor aponeurotic space by 30 ml of 0.75% ropivacaine. The discrepancies of timing and dose may affect the final result, as this volume of local analgesia is sufficient in blocking the posterior branch of obturator nerve joins the canal.^[56]^ Thus, little impact on the result will be of note.

In this meta-analysis, the CACB group had lower VAS scores in the early stage of post-operation (<48 hours) at rest compared to the SACB group, but no statistical significance was observed at 48 hours. In this forest plot, the *I*^2^ was greater than or equal to 50%, which means that the heterogeneity test demonstrates a statistical significance. From the related trials,^[30,62–65]^ several reasons may have led to its heterogeneity, including ethnic differences. Four trials were located in Asia, and 5 took place in Europe or America. Another reason could be that the mean age was different in some studies. Third, the VAS scores may be affected by bilateral TKA or unilateral TKA, but most of the articles included did not mention. Fourth, of the included RCTs, the estimation of the variance and mean from the size of a sample (range = maximum–minimum), as well as the range and the median is necessary. In the article by Hozo et al,^[66]^ for one of the samples (n > 25), the median may be used to assess the mean. If the sample size is larger than 70, the formula range/6 gives the best estimator for the standard deviation concerning a sized moderate sample (15 < n ≤ 70), and the standard deviation is better estimated by the formula range/4. Thus, the results of this study may be influenced.

Moreover, Canbek et al^[40]^ disclosed that the effects of CACB were distinctively superior to SACB than others. The reasons for this discrepancy are as follows. First, to ascertain the correct position of the needle tip in the adductor canal, Canbeks team used an injection of 10 ml saline for verification before delivering analgetics, which may have diluted the local analgetic concentration, leading to poorer pain control in the SACB group. Second, the higher concentration of analgetics was due to additional analgetics being repeatedly given via catheter in the CACB group, further widening the gap in regard to effects of treatment. Moreover, as his study had a relatively large sample size, small differences would represent a larger proportion in the data analysis.

### 4.2 Complications and cumulative morphine consumption

Concerning postoperative pain, an ideal strategy of analgesia is to reduce pain intensity and morphine consumption without increasing the incidence of complications.^[67]^ This meta-analysis showed no significant difference between CACB and SACB in the 2 aspects. Both methods might lessen the pain after TKA, leading to a reduction in the consumption of morphine. ACB is a type of nerve block, however, this study revealed that peripheral nerve blocks may confer a few unusual complications like catheter site infection, nerve injury, and healing ulcers.^[68]^ Additionally, Wang et al^[69]^ reported no significant differences between ACB and FNB in regard to complications with no heterogeneity, such as urinary retention and pruritus.

### 4.3 LOS

LOS describes the economical expenditure of each patient. Owing to both of the groups for effective pain control, it would shorten the patients LOS. LOS is dependent on both patient recovery as well as the effectiveness of pain control. Zhang et al^[41]^ suggested decreased quadriceps strength in patients who received CACB compared to patients who were treated with SACB. Furthermore, these results were deemed to be caused by a blockade of the motor branch of the vastus medial nerve and because of the spread of local analgesia to the femoral triangle in those treated with CACB. Contrarily, Turner et al^[42]^ reported improvements in straight leg raising tests in patients who received SACB compared to patients treated with CACB. These are essential factors that can affect the length of stay, but considerable heterogeneity exists in our meta-analysis. Therefore, the reported outcomes should be carefully considered.

### 4.4 Rescue analgesia

Shah et al^[42]^ reported 2 patients who utilized rescue analgesia in the single-shot group, while no patients used rescue analgesia in the continuous-injection group. Lee et al^[43]^ also reported the additional consumption of opioids in the CACB group. They believed that secondary block failure and catheter displacement may have influenced their results. Moreover, Canbek et al^[40]^ showed no patients were affected by catheter displacement, however, 6 patients were given rescue analgesia in the SACB group with 1 patient in the continuous-injection group. Canbek et al^[40]^ proposed a lower need for rescue analgesia in the continuous-injection group compared to the single-shot group. Li et al^[44]^ demonstrated that more patients demand rescue analgesia compared to those reported by Shah et al.^[45]^ This may be due to the local infiltration of analgesia or intravenous patient-controlled analgesia (IV-PCA) not being used for assisted analgesia in their studies. Additionally, it is not difficult to find that each RCT performed the operation with various doses of analgetics. The lack of a standardised measure makes it difficult to interpret these results with confidence, so caution must be applied. Nonetheless, this was an important part of the evaluation analgesic effects. We propose a direction here, which can be further studied in the future.

We also found that there were some inconsistent results from the 2 previous meta-analyses. First, the research by Zhang et al included only 4 RCTs with a total of 322 patients.^[51]^ The results showed that the patients who received CACB had a better efficacy in VAS scores at 48 hours (both of rest-VAS and mobilization-VAS) than those who underwent SACB. However, the SACB group had similar efficacy compared with the CACB group in terms of morphine consumption, time to first opioid request, range of motion, and VAS scores(both of rest-VAS and mobilization-VAS) at 24 hours and 48 hours, also without increasing the risk of complications and length of stay. Therefore, the team concluded that the SACB may be more preferable for hospitals without experienced anesthesiologists and resources to perform the continuous infusions, compared with the CACB method. The limited studies (only 4 RCTs) included in their analysis very likely contributed to these inconsistencies. Additionally, the number of sample size (739 patients) in our study is more than double its number in the study (Zhang, 2019), which would lead to significant differences in the final results. Second, the study conclusions of 642 patients a recent meta-analysis conducted by Wang et al.^[70]^ However, there are still differences in some results. Wang's team included the RCT by Zhang et al^[41]^ in terms of rest-VAS scores at 48 hours, mobilization-VAS at 24 hours and morphine consumption, in our opinion, which is unreasonable. The RCT reported those results of the terms via bar charts without specific and accurate data, so it was difficult to get accurate data only a rough approximation. In general, nevertheless, Wang's work is a systematic and comprehensive analysis.

### 4.5 Limitations

This meta-analysis possesses several limitations. Only 9 RCTs were included in the study, and the sample size is small. Due to insufficient data, we were unable to perform a meta-analysis for postoperative knee function, which is a significant parameter. Furthermore, because of the lack of sufficient extracted data and comparability between the included articles, some outcomes could not be analyzed. The underestimation of complications may be related to short-term follow-up. Publication bias may exist due to insufficient data of the included studies.

## 5 Conclusion

The present meta-analysis indicated that CACB may be superior to SACB in items of analgesic effect after TKA. However, due to the limitations of the included studies, the conclusions from this research should be carefully considered. In this regard, additional high-quality and large-sample clinical trials are necessary to certify the efficacy and safety of CACB compared to SACB following TKA.

## Acknowledgments

We are very grateful for many helpful comments on an earlier version of this manuscript.

## Author contributions

Rongguo Yu and Yiyuan Zhang performed study design. Rongguo Yu was responsible for manuscript review.

**Conceptualization:** Rong-guo Yu, Yiyuan Zhang.

**Data curation:** Rong-guo Yu, Hai-yang Wang, Dong-xin Liu.

**Formal analysis:** Rong-guo Yu, Hai-yang Wang, You-guang Zhuo, Dong-xin Liu.

**Investigation:** Yiyuan Zhang.

**Methodology:** Rong-guo Yu, Hai-yang Wang, You-guang Zhuo, Chun-ling Wu.

**Resources:** Yiyuan Zhang.

**Software:** Rong-guo Yu, You-guang Zhuo.

**Supervision:** Yiyuan Zhang.

**Visualization:** Rong-guo Yu.

**Writing – original draft:** Rong-guo Yu, Hai-yang Wang, Dong-xin Liu.

**Writing – review & editing:** Rong-guo Yu, Hai-yang Wang, You-guang Zhuo.

## Correction

Affiliation a appeared incorrectly as “Department of Orthopedics, Fuzhou the second Hospital Affiliated to Xiamen University, Fujian” and has been corrected to “Department of Orthopedics, Fuzhou second Hospital Affiliated to Xiamen University, Fujian”.
